# The dose–response relationship between physical activity and the risk of death from pneumonia in middle-aged and older adults: A meta-analysis

**DOI:** 10.1097/MD.0000000000038220

**Published:** 2024-05-24

**Authors:** Songtao Lu, Zhiqi Shuai, Yunfei Lu

**Affiliations:** a School of Sports and School of Physical Education, Wuhan University of Science and Technology, Wuhan, Hubei Province, China; b School of Sports and School of Physical Education, Central China Normal University, Wuhan, Hubei Province, China.

**Keywords:** meta-analysis, middle-aged, mortality, older adults, physical activity, pneumonia

## Abstract

**Background:**

Deaths from COVID-19 are concentrated in older adults, and studies have reported that physical activity (PA) can reduce the risk of death from pneumonia.

**Methods:**

Eight cohort studies and 2 case–control studies were included according to the inclusion and exclusion criteria established in this meta-analysis study followed the PRISMA guideline, 8 cohort studies and 2 case–control studies were finally included. Then, the research objects in these studies were classified to further study the dose–response relationship and non-dose–response relationship.

**Results:**

The highest dose of PA reduced the risk of death by 59% (risk ratio = 0.41; 95% confidence interval: 0.23–0.58) compared with the lowest dose of PA in middle-aged and elderly people. Furthermore, when the PA level was <10 m/wk, the risk of death from pneumonia was reduced by 6% every 4.5 MET-h/wk increase. At a PA level > 10 m/wk, the risk of death from pneumonia increased by 5% every 4.5 MET-h/wk increase. At a PA level > 30 m/wk, PA is a risk factor for pneumonia-related death in middle-aged and elderly people.

**Conclusions:**

This meta-analysis showed that PA was associated with a reduced risk of dying from pneumonia in middle-aged and older adults, and that there was a significant nonlinear negative dose–response relationship between PA levels and the risk of dying from pneumonia. Therefore, moderate exercise was recommended.

## 1. Introduction

Pneumonia refers to an infection of the lungs caused by pathogens, such as bacteria and viruses, often involving typical symptoms, such as fever, cough, and dyspnea. Pneumonia can be classified as community-acquired or hospital-acquired pneumonia according to the place of origin of the infection.^[1]^ In December 2019, a pneumonia epidemic caused by a new unknown virus emerged in Wuhan, China. The World Health Organization (WHO) named the new coronavirus pneumonia “COVID-19” (coronavirus disease 2019) on February 11.^[2]^ At the same time, the International Committee on the Classification of Viruses (ICOV) stated that the new coronavirus would be named “SARS-CoV-2” (severe acute respiratory syndrome coronavirus 2). Owing to the rapid spread of COVID-19 worldwide and the high severity of the disease, the epidemiological situation was of great concern, and following an assessment, the WHO declared the COVID-19 epidemic as a pandemic on March 11, 2020. According to WorldOmeter data, as of May 23, 2023, the cumulative number of confirmed cases worldwide exceeded 689 million, and the cumulative number of deaths worldwide exceeds 6.88 million. Since the start of the pandemic, there have been 114 countries or regions with more than 2000 cumulative deaths, totaling 6,579,788 cumulative deaths, accounting for 99.3% of the cumulative deaths worldwide. Various public health interventions, such as mask-wearing and vaccination, have been implemented globally to mitigate and control the spread of COVID-19.^[3]^ Serious illnesses requiring hospitalization and death due to pneumoconiosis occur mainly in older adults. Middle-aged and older adults are more likely to have underlying illnesses and to be less fit; therefore, they are more likely to develop severe pneumonia or respiratory and circulatory dysfunction, leading to irreversible sepsis and metabolic acidosis, all of which are significantly more difficult to treat and have a correspondingly higher mortality rate.

Physical activity (PA) has been widely recognized as an effective means of reducing the risk of noncommunicable diseases, including cancer, cardiovascular disease, and other chronic verifiable diseases.^[4]^ Available evidence suggests that PA can reduce the risk of contracting a range of infectious diseases, including those caused by viruses and bacteria. A recent US study published in the online edition of the British Journal of Sports Medicine showed that regular aerobic exercise significantly reduced the risk of death from influenza or pneumonia. A previous systematic review and meta-analysis by Rahmati et al^[5]^ reported that increased PA levels reduced hospitalization rates, intensive care unit (ICU) admissions, and mortality in patients with COVID-19 across all study types. A meta-analysis by Sittichai et al^[6^^]^ also showed that regular PA reduced the severity and risk of death in COVID-19 patients, particularly ≥150 min/wk of moderate activity or ≥75 min/wk of vigorous activity. There are many reviews and meta-analyses of studies on PA and mortality risk, mainly focusing on all-cause mortality and cancer mortality. Studies by Ukawa et al ^[7]^ have shown that both low- and moderate-to-high-intensity PA are protective factors, with a significantly lower risk of pneumonia-related death relative to the lowest dose of PA. Walking is a PA that is common in older adults and has been shown to have several health benefits, including a reduced risk of pneumonia-related mortality.^[8]^ Meta-analyses of the association between PA and the risk of mortality from pneumonia are also slowly emerging. However, meta-analyses focusing on the dose–response relationship between the risk of pneumonia mortality and PA in middle-aged and older adults have not been performed. Therefore, this study attempted to conduct a meta-analysis to quantify the association between different doses of PA and the risk of pneumonia mortality in middle-aged and older adults, and this study covers pneumonia in both COVID and non-COVID-19 populations.

## 2. Methods

### 2.1. Search strategy

Following the PRISMA guidelines, the researchers systematically searched electronic databases (from 1980 to date), including PubMed, Medicine, Web of Science, and others, for literature on the association between PA and pneumonia mortality. We searched for the literature using the keywords (SARS-CoV-2 or COVID-19 or pneumonia) and (PA or exercise or physical training) and (mortality or death) and (middle-aged or senior or older adults), with a series of articles and relevant review articles. In addition, we searched the reference lists of all included studies to identify other eligible articles. No search language restrictions were applied.

### 2.2. Statistical analyses

Meta-analysis was performed using STATA software (version 16.0; *P* values < .05), and all tests were double-sided. We performed separate meta-analyses for categorical and continuous variables to assess the association between PA and the risk of mortality from pneumonia. Pooled results were expressed as risk ratios (RRs) with 95% confidence intervals (CIs). Pooled analyses were categorized into cohort and cross-sectional studies based on study type, and pooled RRs were estimated using a random-effects model. Heterogeneity was assessed and described using the *I*^2^ statistic as the percentage of variation in the study, with *I*^2^ values of 25%, 50%, and 75% indicating low, medium, and high levels of heterogeneity, respectively.^[9]^ A cutoff 0.1 is for the *P* value to assessing heterogeneity. Egger and Begg tests were used to determine whether publication bias existed.^[10]^ For the sensitivity analysis, each study was removed individually to check whether the combined effect of the remaining studies had changed.

In the continuous variable analysis, the MET values were included to reflect different levels of PA. We converted the duration of a given PA intensity (h/wk) to MET-h/wk by combining it with the weekly frequency. We classified as LPA (3 MET-h, e.g., walking exercise), MVPA (4.5 MET-h), and VPA (8 MET-h).^[11]^ The dose metabolic equivalent (MET) used in this study was a physiological index describing the metabolic equivalent of PA energy in humans, defined as the energy consumed per kilogram of body weight per hour: 1 MET = 1 kcal/kg*h.

A robust error meta-regression method described by Xu and Doi^[12]^ was used to calculate continuous dose–response relationship slopes (nonlinear trends) and 95% CIs from the natural logs of the reported RRs and CIs across categories of LTPA measures. This method is based on a one-stage approach that considers each study as a cluster of the whole sample and treats the within-study correlations using clustered robust errors. Based on the goodness of fit test of the model, the Stata software XBLC command was used to draw a dose–response curve.^[13]^

### 2.3. Selection of studies

The following 4 inclusion criteria were used in the current meta-analysis: published observational cohort studies or case–control studies; studies in which the participants were middle-aged and older adults over 45 years of age; and studies that reported the odds ratios, relative risks (RRs or HRs), and 95% confidence intervals (95% CIs) of PA levels related to the risk of mortality from pneumonia (or provided raw data to calculate these indicators). If multiple articles were published in the same group, those with longer tracking times or larger sample sizes were selected. The researchers searched the literature according to the above criteria separately, identified the studies that MET the requirements, and then discussed the studies together to determine whether they should be included.

### 2.4. Quality assessment

The Newcastle-Ottawa Scale (NOS) was used to assess the quality of the literature. The higher the overall score, the higher was the quality of the study, with a maximum score of 9. Studies with NOS scores of 0 to 3, 4 to 6, and 7 to 9 were considered low, moderate, and high quality, respectively.^[14]^ If there was an inconsistent evaluation of the quality of the literature, the group focused on this issue and determined a final score based on quality.

## 3. Results

### 3.1. Literature search and study characteristics

**Figure 1.**
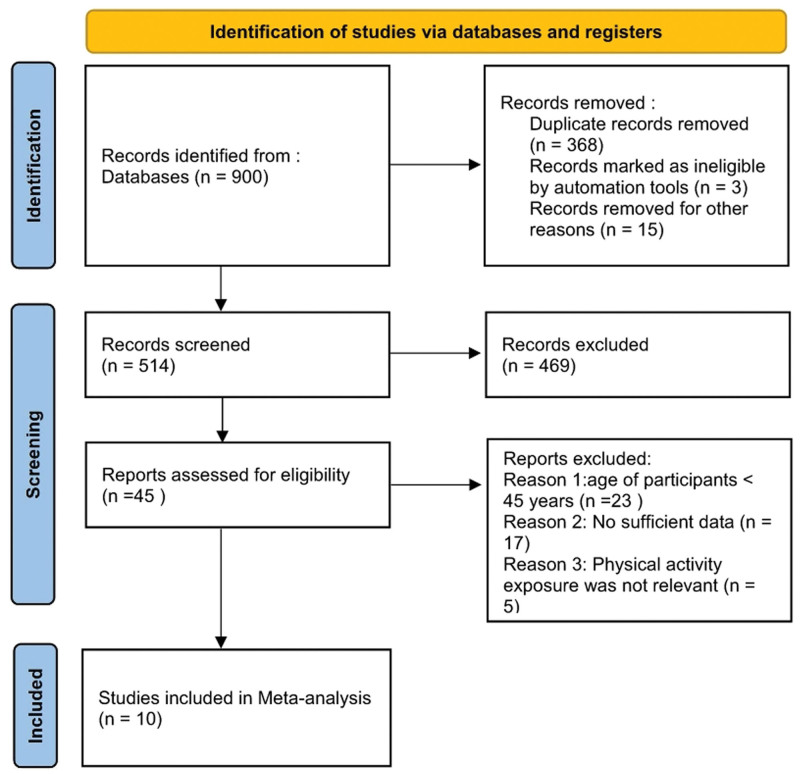
Flow diagram of studies considered for inclusion in the systematic review.

A total of 900 potentially relevant citations were identified through the database and citation searches. We identified 558 articles in PubMed, 35 articles in Medicine, 274 articles in Web of Science, and 33 articles in GeenMedica. According to the inclusion and exclusion criteria established in this study, 8 cohort studies and 2 case–control studies^[15–25]^ involving a total of 1,237,326 participants and approximately 3646 pneumonia-related deaths were ultimately included. Table 1 summarizes the characteristics of each study. Two of the 10 studies were from the US, 1 was from Asia, and 7 were from Europe. Seven of these articles were rated as high-quality studies and the rest were rated as moderate-quality studies. A flow chart of the literature search and study inclusion process is shown in Figure 1.

**Table 1:** Characteristics of included studies.

Study (y)	Country	Research type	Case/total	Age	Dose of PA	Research quality
Ahmadi et al^[15]^ (2021)	UK	Cohort	387/468,569	56.5 ± 8.1	3 doses: high, medium, and low	8
Ryrsø et al^[24]^ (2022)	Denmark	Cohort	60/326	71 (mean)	2 doses: medium to Vigorous and low	8
Cho et al^[22]^ (2021)	Korean	Case-control	75/125,780	50.7 ± 14.3	5 doses: MET-min/wk	9
Ekblom-Bak et al^[23]^ (2021)	Sweden	Case-control	138/279,455	49.9 (mean)	3 doses: exercise times	7
Hamer et al^[16]^ (2019)	UK	Cohort	1270/97,844	47.1 (mean)	3 doses: inactive, Insufficient, and sufficiently active	8
Hamrouni et al ^[18]^ (2021)	UK	Cohort	397/259,397	65 (mean)	3 doses: high, medium, and low	8
Myint et al^[21]^ (2016)	UK	Cohort	503/2465	59 ± 18	2 dose: inactive and active	6
Salgado-Aranda et al^[20]^ (2021)	Spain	Cohort	45/552	54.3 ± 10.7	2 dose: sedentary and physically	6
Sallis et al^[19]^ (2021)	USA	Cohort	771/2970	47 ± 16.97	3 doses: high, medium, and low	9
Salive et al^[17]^ (1993)	USA	Cohort	NP	58.6 (mean)	NP	6

PA = physical activity.

### 3.2. *Categorical dose–response relationship between PA and the risk of death from pneumonia in middle-aged and older adults*

**Figure 2.**
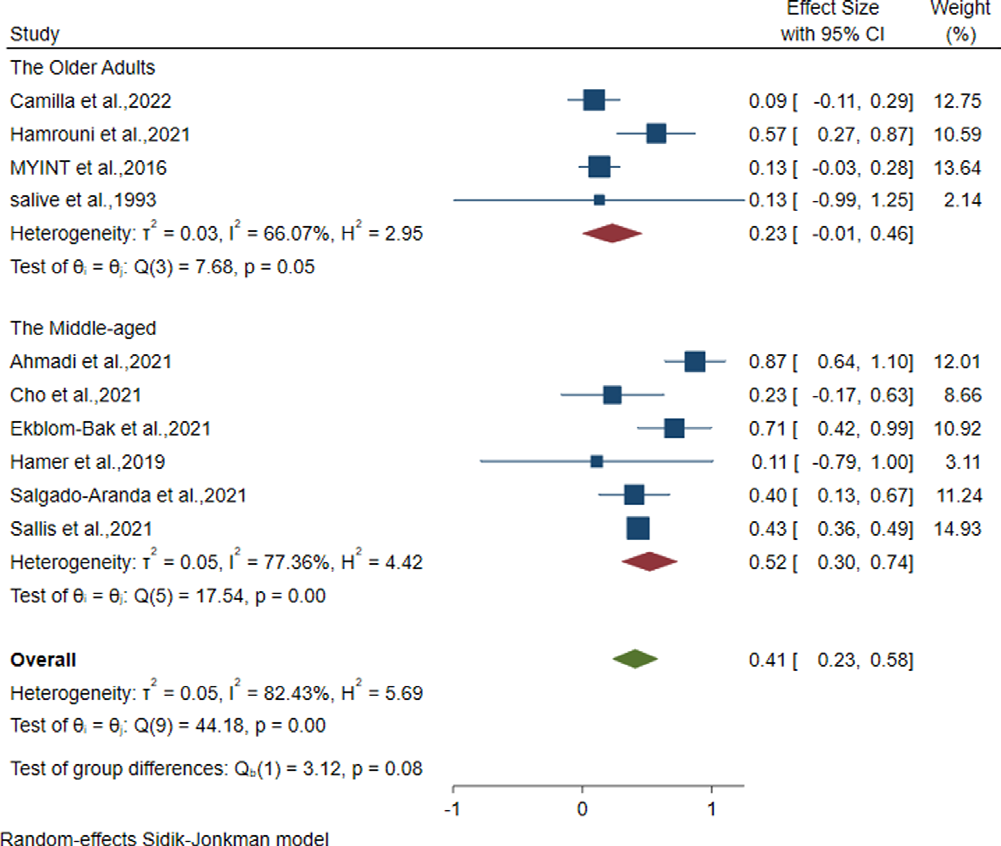
Categorical dose–response relationship between PA and the risk of death from pneumonia in middle-aged and older adults. PA = physical activity.

A pooled RR model was used to assess the effect of PA on mortality due to pneumonia in middle-aged and older adults. Compared to the lowest dose of PA, the highest dose of PA reduced the risk of pneumonia mortality by 59% (RR = 0.41; 95% CI: 0.23–0.58), and the test of heterogeneity resulted in an I^2^ statistic of 82.43% (*P* = .000), indicating significant heterogeneity in the study results (Fig. 2). In the sensitivity analysis, the overall pooled estimates of the respective results obtained in each analysis were very similar to those of the preliminary associations. In addition, we performed a funnel plot examination of the included studies, which showed no significant bias.

As shown in Table 2, the *P* value for the test of heterogeneity between subgroups for age, different doses of PA, and BMI data were <0.1 according to meta-random-effects model analysis, indicating significant heterogeneity between studies. This indicates that age is an important factor affecting the relationship between PA and pneumonia mortality. Tests for heterogeneity within subgroups showed that the study type, age, study quality, and different doses of PA, with adjustment for sex, BMI, hypertension, cardiovascular disease, diabetes, and cancer, all had *P* values of <0.1, indicating great heterogeneity in the studies. It is worth mentioning that the middle-aged subgroup (those aged between 45 and 60 years) showed significant heterogeneity (*P* = .000), while the older adult subgroup over 60 years of age also showed significant heterogeneity (*P* < .1).

**Table 2:** Subgroup analysis.

Subgroup	n	RR (95% CI)	*I*^2^ (%)	*P* within subgroup	*P* between subgroup
Study type					
Cohort	8	0.38 (0.18, 0.58)	84.45	.000	.67
Case–control	2	0.49 (0.04, 0.94)	70.83	.032	
Age					
≦60	6	0.52 (0.30, 0.74)	77.36	.000	.08
>60	4	0.23 (−0.01, 0.46)	66.07	.055	
Study quality					
High	7	0.47 (0.24, 0.69)	84.07	.000	.11
Moderate	3	0.22 (0.03, 0.42)	30.64	.027	
Dif dose					
Multi	6	0.55 (0.33, 0.77)	75.71	.000	.01
Binary	4	0.18 (0.02, 0.34)	37.54	.027	
Dif country					
Europe	7	0.43 (0.20, 0.67)	82.76	.000	.65
Asia	1	0.23 (−0.17, 0.63)		.254	
America	2	0.42 (0.27, 0.58)	3.21	.000	
Adjusts sex					
Yes	7	0.47 (0.25, 0.69)	85.37	.000	.12
No	3	0.22 (−0.01, 0.44)	33.35	.059	
Adjust stage					
Yes	8	0.46 (0.25, 0.67)	80.49	.000	.12
No	2	0.24 (−0.01, 0.49)	63.06	.062	
Adjust BMI					
Yes	5	0.60 (0.38, 0.82)	74.32	.000	.00
No	5	0.18 (0.04, 0.32)	26.52	.010	
Adjust hypertension					
Yes	3	0.53 (0.17, 0.88)	87.68	.004	.36
No	7	0.34 (0.14, 0.54)	67.75	.001	
Adjust cardiovascular					
Yes	5	0.45 (0.19, 0.70)	89.65	.001	.60
No	5	0.35 (0.09, 0.61)	59.20	.008	
Adjust diabetes					
Yes	3	0.47 (0.06, 0.88)	95.99	.025	.68
No	7	0.37 (0.17, 0.57)	54.75	.000	
Adjust cancer					
Yes	2	0.63 (0.22, 1.05)	91.51	.003	.18
No	8	0.32 (0.14, 0.50)	62.62	.000	

**Figure 3.**
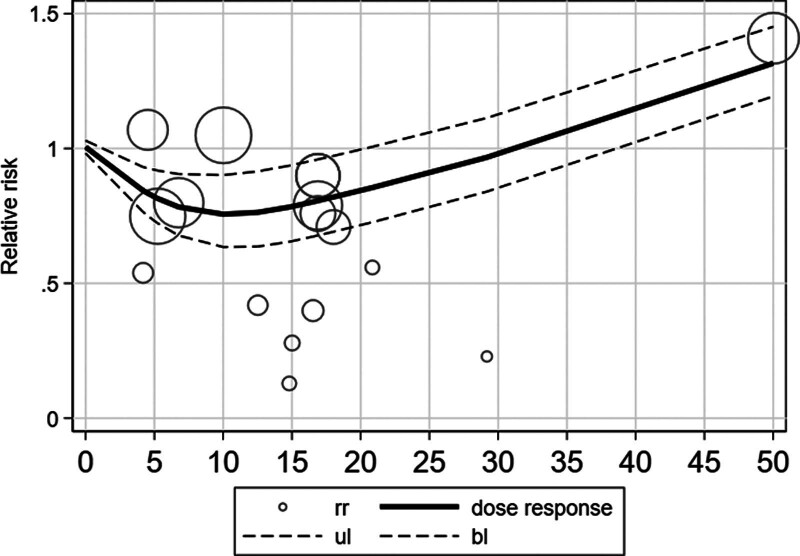
Continuous dose–response relationship between PA and the risk of death from pneumonia in middle-aged and older adults. PA = physical activity.

In the sensitivity analyses, the overall pooled estimates of the respective outcomes obtained in each analysis were very similar to the preliminary correlations. In addition, inspection of the funnel plots of the included studies indicated no significant bias in this meta-analysis (Fig. 3). In addition, there was no significant publication bias in this study (Begg test *P* value = 1.142 > .05 and Egger test *P* value = .671 > .05).

### 3.3. Continuous dose–response relationship between PA and the risk of death from pneumonia in middle-aged and older adults

**Figure 4.**
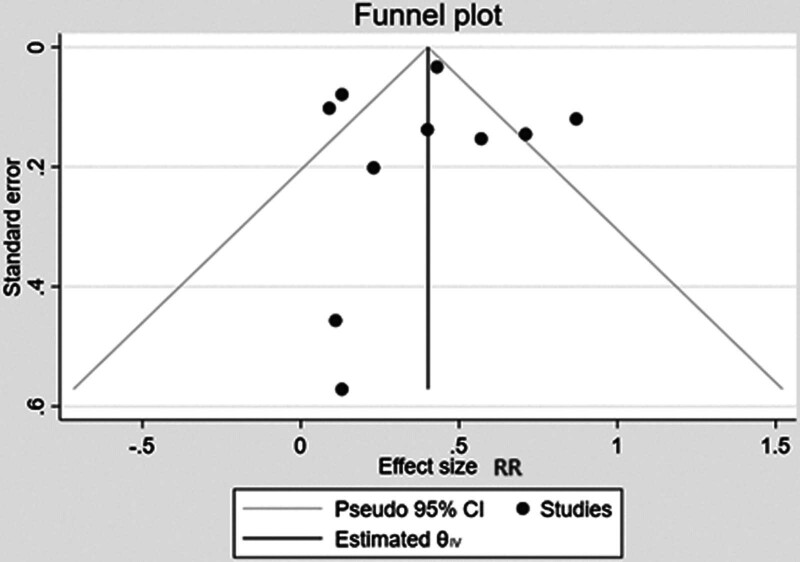
Funnel plots for publication bias.

Figure 4 shows the continuous dose–response relationship between PA and the risk of death from pneumonia in middle-aged and older adults. The results showed a negative nonlinear relationship between PA and pneumonia mortality in middle-aged and older adults. At a PA level < 10 MET-h/wk, the risk of dying from pneumonia was reduced by 6% for every additional 4.5 MET-h/wk. When the PA level was >10 MET-h/wk, the risk of dying from pneumonia increased by 5% for every additional 4.5 MET-h/wk. However, at a PA level > 30 m/wk, PA is a risk factor for pneumonia-related death in middle-aged and elderly people. Therefore, PA cannot be excessive, and excessive PA is harmful for middle-aged and older adults.

## 4. Discussion

The results of this meta-analysis suggest that PA may be an important protective factor in reducing mortality due to pneumonia in middle-aged and older adults. Specifically, point estimates of log-risk ratios for all included studies indicated that the most physically active group was less likely to die than the least physically active group. Regular PA has been well-documented to reduce the risk of several chronic diseases and mortality. Although PA guidelines recommend 150 to 300 min/wk of moderate-intensity activity or 75 to 150 min/wk of high-intensity aerobic PA/exercise for adults, most populations do not reach these levels. Only 28% of urban residents in the United States reportedly follow the exercise recommendations in these guidelines.

Several studies have confirmed the ability of PA to reduce the risk of pneumonia mortality in older adults. A large cohort study by Ukawa et al ^[7]^ showed that walking for ≥ 1 h/d reduced the risk of pneumonia mortality in older adults with or without a history of MI, and their findings suggested that regular walking may be beneficial in reducing the risk of pneumonia mortality in older adults. Kunutsor et al^[25]^ reported that regular PA was associated with a lower risk of incident pneumonia and pneumonia-related mortality in the general population. Studies have also demonstrated a higher risk of pneumonia and its associated consequences in sedentary older adults. Elezkurtaj et al^[26]^ showed that the risk of death is associated with lower baseline physical performance. Therefore, there is a need to identify an attractive and feasible type of PA or exercise for middle-aged and older adults to ensure regular PA. Although any form of PA is beneficial to the health of middle-aged and older people, care should be taken not to perform too much activity, as this may lead to sports injuries. For people aged ≥ 65 years, the benefits of PA are reflected in the following health outcomes: improved all-cause mortality, cardiovascular mortality, mental health (reduced anxiety and depressive symptoms), cognitive health and sleep, and improvements in obesity indices.

Through a meta-analysis of categorized doses, our primary conclusion was that the risk of death from pneumonia in middle-aged and older adults was reduced by 59% in those with the highest dose of PA compared to those with the lowest dose of PA. Significant heterogeneity between age subgroups indicates that age is an important factor affecting the relationship between PA and pneumonia mortality. Through continuous dose–response analysis, we confirmed that the relationship between PA and the risk of dying from pneumonia in middle-aged and older adults was nonlinear and inverse. For every 4.5 MET-h/wk increase in PA, the risk of dying from pneumonia in middle-aged and older adults was reduced by 5% to 6%. At a PA level > 30 m/wk, PA is a risk factor for pneumonia-related death in middle-aged and elderly people. The WHO global recommendations on the health benefits of PA state that adults should perform at least 150 minutes of moderate-intensity aerobic PA per week and at least 75 minutes of high-intensity aerobic PA per week, or a combination of moderate-intensity and vigorous-intensity activity.^[27]^

Finally, our study is the first meta-analysis of the dose–response relationship between PA and the risk of pneumonia mortality in middle-aged and older adults. The results of this study, which are relatively stable, are based on large cohort and case–control studies. However, this study had several limitations. First, the number of included studies may not have been sufficient. This study strictly followed the inclusion and age criteria for middle-aged and older adults, and there were few studies that MET all the criteria. Second, the PA assessment methods in the literature included in this study were subjective measures, which may lead to inaccurate doses and different methods of measuring and observing PA and may also have an impact on the results of the study. Third, some of the included cohort studies did not adjust for confounding factors such as cardiovascular disease, diabetes, hypertension, and other comorbid conditions. In addition, the definition of PA intensity varied among the studies and should be consistent in future studies. Physical activity was self-reported; therefore, there may be misclassification bias, which can be avoided in future studies using objective PA assessments (e.g., accelerometer-based data).

## 5. Conclusion

This meta-analysis showed that PA was associated with a reduced risk of dying from pneumonia in middle-aged and older adults and that there was a significant nonlinear negative dose–response relationship between PA levels and the risk of dying from pneumonia. Encouraging older adults to be physically active throughout their lives may be a viable and safe way to slow the increase in pneumonia mortality.

## Acknowledgments

The authors thank all the reviewers for their helpful comments.

## Author contributions

**Conceptualization:** Songtao Lu, Yunfei Lu, Zhiqi Shuai.

**Methodology:** Songtao Lu, Zhiqi Shuai.

**Resources:** Songtao Lu.

**Software:** Songtao Lu, Yunfei Lu, Zhiqi Shuai.

**Visualization:** Songtao Lu, Yunfei Lu, Zhiqi Shuai.

**Writing – original draft:** Songtao Lu, Yunfei Lu.

**Writing – review & editing:** Songtao Lu, Yunfei Lu, Zhiqi Shuai.

**Formal analysis:** Yunfei Lu.

**Validation:** Zhiqi Shuai.

## References

[1] Cilloniz C Martin-Loeches I Garcia-Vidal C San Jose A Torres A. Microbial etiology of pneumonia: epidemiology, diagnosis and resistance patterns. Int J Mol Sci. 2016;17:2120. 27999274 10.3390/ijms17122120 PMC5187920

[2] World Health Organization. Newsroom. Geneva, Switzerland: World Health Organization; 2010. Available at: https://www.who.int/news-room/fact-sheets/detail/coronavirus-disease-(covid-19) [access date January 12, 2024].

[3] Parodi SM Liu VX. From containment to mitigation of COVID-19 in the US. JAMA. 2020;323:1441–2. 32167525 10.1001/jama.2020.3882

[4] Nieman DC Henson DA Austin MD Sha W. Upper respiratory tract infection is reduced in physically fit and active adults. Br J Sports Med. 2010;45:987–92. 21041243 10.1136/bjsm.2010.077875

[5] Rahmati M Shamsi MM Khoramipour K. Baseline physical activity is associated with reduced mortality and disease outcomes in COVID-19: a systematic review and meta-analysis. Rev Med Virol. 2022;13:e2349. 10.1002/rmv.2349 PMC9111124 35416354

[6] Sittichai N Parasin N Saokaew S. Effects of physical activity on the severity of illness and mortality in COVID-19 patients: a systematic review and meta-analysis. Front Physiol. 2022;13:1030568. 36439253 10.3389/fphys.2022.1030568 PMC9686861

[7] Ukawa S Zhao W Yatsuya H. Associations of daily walking time with pneumonia mortality among elderly individuals with or without a medical history of myocardial infarction or stroke: findings from the Japan collaborative cohort study. J Epidemiol. 2019;29:233–7. 30249944 10.2188/jea.JE20170341 PMC6522391

[8] Ikeda T Inoue S Konta T. Can daily walking alone reduce pneumonia-related mortality among older people? Sci Rep. 2020;10:8556. 32444618 10.1038/s41598-020-65440-z PMC7244731

[9] Higgins J Thompson S. Quantifying heterogeneity in a meta-analysis. Stat Med. 2002;21:1539–58. 12111919 10.1002/sim.1186

[10] Begg CB Mazumdar M. Operating characteristics of a rank correlation test for publication bias. Biometrics. 1995;50:1088–101. 7786990

[11] Ainsworth B Haskell W Herrmann S. 2011 compendium of physical activities: a second update of codes and MET values. Med Sci Sports Exerc. 2011;43:1575–81. 21681120 10.1249/MSS.0b013e31821ece12

[12] Xu C Doi SAR. The robust error meta-regression method for dose–response meta-analysis. Int J Evid Based Healthc. 2018;16:138–44. 29251651 10.1097/XEB.0000000000000132

[13] Zhang T Dong S Li B.. Advanced Meta Analysis Method: A Stata-Based Implementation. Vol. 10. ShangHai: Fudan University Press; 2015. p. 220–232.

[14] Wells GA Peterson J Welch V. The Newcastle-Ottawa Scale (NOS) for Assessing the Quality in Nonrandomized Studies in Metaanalyses. Ottawa: University of Ottawa; 2009. Available at: https://www.ohri.ca/programs/clinical_epidemiology/oxford.asp.

[15] Ahmadi MN Huang BH Inan-Eroglu E Hamer M Stamatakis E. Lifestyle risk factors and infectious disease mortality, including COVID-19, among middle aged and older adults: evidence from a community-based cohort study in the United Kingdom. Brain Behav Immun. 2021;96:18–27. 33940153 10.1016/j.bbi.2021.04.022 PMC8127518

[16] Hamer M O’Donovan G Stamatakis E. Lifestyle risk factors, obesity and infectious disease mortality in the general population: linkage study of 97,844 adults from England and Scotland. Prev Med. 2019;123:65–70. 30844499 10.1016/j.ypmed.2019.03.002

[17] Salive ME Satterfield S Ostfeld AM Wallace RB Havlik RJ. Disability and cognitive impairment are risk factors for pneumonia-related mortality in older adults. Public Health Rep. 1993;108:314–22. 8497569 PMC1403382

[18] Hamrouni M Roberts M Thackray A Stensel D Bishop N. Associations of obesity, physical activity level, inflammation and cardiometabolic health with COVID-19 mortality: a prospective analysis of the UK Biobank cohort. BMJ Open. 2021;11:e055003. 10.1136/bmjopen-2021-055003 PMC8572360 34732503

[19] Sallis R Young DR Tartof SY. Physical inactivity is associated with a higher risk for severe COVID-19 outcomes: a study in 48 440 adult patients. Br J Sports Med. 2021;55:1099–107. 33849909 10.1136/bjsports-2021-104080

[20] Salgado-Aranda R Perez-Castellano N Nunez-Gil I. Influence of baseline physical activity as a modifying factor on COVID-19 mortality: a single-center, retrospective study. Infect Dis Ther. 2021;10:801–14. 33715099 10.1007/s40121-021-00418-6 PMC7955903

[21] Myint PK Hawkins KR Clark AB. Long-term mortality of hospitalized pneumonia in the EPIC-Norfolk cohort. Epidemiol Infect. 2016;144:803–9. 26300532 10.1017/S0950268815001971 PMC6217917

[22] Cho DH Lee SJ Jae SY. Physical activity and the risk of COVID-19 infection and mortality: a nationwide population-based case–control study. J Clin Med. 2021;10:1–11. 10.3390/jcm10071539 PMC8038831 33917558

[23] Ekblom-Bak E Vaisanen D Ekblom B. Cardiorespiratory fitness and lifestyle on severe COVID-19 risk in 279,455 adults: a case control study. Int J Behav Nutr Phys Act. 2021;18:135. 34666788 10.1186/s12966-021-01198-5 PMC8524225

[24] Ryrsø CK Hegelund MH Dungu AM. Association between Barthel index, grip strength, and physical activity level at admission and prognosis in community-acquired pneumonia: a prospective cohort study. J Clin Med. 2022;11:6326–37. 36362554 10.3390/jcm11216326 PMC9653820

[25] Kunutsor SK Seidu S Laukkanen JA. Physical activity reduces the risk of pneumonia: systematic review and meta-analysis of 10 prospective studies involving 1,044,492 participants. Geroscience. 2022;44:519–32. 34822066 10.1007/s11357-021-00491-2 PMC8811019

[26] Elezkurtaj S Greuel S Ihlow J. Causes of death and comorbidities in hospitalized patients with COVID-19. Sci Rep. 2021;11:1–9. 33608563 10.1038/s41598-021-82862-5 PMC7895917

[27] Piercy KL Troiano RP Ballard RM. The physical activity guidelines for Americans. JAMA. 2018;320:2020–8. 30418471 10.1001/jama.2018.14854 PMC9582631

